# Mechanisms of Genome Maintenance in Plants: Playing It Safe With Breaks and Bumps

**DOI:** 10.3389/fgene.2021.675686

**Published:** 2021-06-22

**Authors:** Aamir Raina, Parmeshwar K. Sahu, Rafiul Amin Laskar, Nitika Rajora, Richa Sao, Samiullah Khan, Rais A. Ganai

**Affiliations:** ^1^Mutation Breeding Laboratory, Department of Botany, Aligarh Muslim University, Aligarh, India; ^2^Botany Section, Women’s College, Aligarh Muslim University, Aligarh, India; ^3^Department of Genetics and Plant Breeding, Indira Gandhi Agriculture University, Raipur, India; ^4^Department of Botany, Bahona College, Jorhat, India; ^5^National Agri-Food Biotechnology Institute, Mohali, India; ^6^Watson-Crick Centre for Molecular Medicine, Islamic University of Science and Technology, Awantipora, India

**Keywords:** DNA damage, DNA repair pathways, mutations, genome integrity, DNA replication

## Abstract

Maintenance of genomic integrity is critical for the perpetuation of all forms of life including humans. Living organisms are constantly exposed to stress from internal metabolic processes and external environmental sources causing damage to the DNA, thereby promoting genomic instability. To counter the deleterious effects of genomic instability, organisms have evolved general and specific DNA damage repair (DDR) pathways that act either independently or mutually to repair the DNA damage. The mechanisms by which various DNA repair pathways are activated have been fairly investigated in model organisms including bacteria, fungi, and mammals; however, very little is known regarding how plants sense and repair DNA damage. Plants being sessile are innately exposed to a wide range of DNA-damaging agents both from biotic and abiotic sources such as ultraviolet rays or metabolic by-products. To escape their harmful effects, plants also harbor highly conserved DDR pathways that share several components with the DDR machinery of other organisms. Maintenance of genomic integrity is key for plant survival due to lack of reserve germline as the derivation of the new plant occurs from the meristem. Untowardly, the accumulation of mutations in the meristem will result in a wide range of genetic abnormalities in new plants affecting plant growth development and crop yield. In this review, we will discuss various DNA repair pathways in plants and describe how the deficiency of each repair pathway affects plant growth and development.

## Introduction

DNA replication is a fundamental process required for all organisms to divide and grow. It encompasses the precise duplication of DNA into two identical copies for the preservation of genetic information (Burgers and Kunkel, 2017). DNA is constantly subjected to numerous diverse kinds of insults that alter its sequence and its chemical nature, affecting the conservation of this information (Carusillo and Mussolino, 2020). The primary source of this alteration is the occasional incorporation of errors during the duplication of DNA by enzymes called DNA polymerases (Ganai and Johansson, 2016). These sporadically incorporated incorrect nucleotides in the newly synthesized DNA occasionally escape the proofreading by the exonuclease site of the DNA polymerases, thereby generating errors (Joyce, 1997). These errors during the process of cell division can have severe consequences on the fitness and viability of an offspring. Remarkably, the errors introduced by DNA polymerase are limited because of the high selectivity by the snugly fit active site of these enzymes and the accompanying ability to excise the incorrect nucleotides (Hogg et al., 2014). In addition to the replication-mediated errors, DNA is constantly exposed to endogenous and exogenous DNA-damaging agents affecting the biochemical and physical properties of the DNA (Aguilera and García-Muse, 2013; Table 1). The mutations arising from these errors can have a catastrophic effect leading to the initiation of genetic and age-related diseases such as cancer and aging. Interestingly, some errors that escape these repair processes can at times act as a source of genetic diversity and pave way for the selection of a better and fitter organism (Karthika et al., 2020).

**TABLE 1:** List of major DNA-damaging agents associated with different DNA repair pathways and their sources.

Repair pathway	DNA damages	Source
Direct reversal repair	6-4PP (dinucleoside monophosphate 6-4 photoproduct)	UV radiation
	CPD (cyclobutane pyrimidine nucleoside phosphate dimer)	UV radiation
	*O*^6^-alkylG (*O*^6^-alkyl-2′-deoxyguanosine-5′-monophosphate)	Alkylating agents
	Pyrimidine dimer (dipyrimidine nucleoside phosphate dimer)	UV radiation
	Thymidine dimer (dithymidine nucleoside phosphate dimer)	UV radiation
	1,*N*6-ethenoA (1,*N*6-etheno-2′-deoxyadenosine-5′-monophosphate)	Vinyl chloride metabolites Chloroethylene oxide Chloroacetaldehyde
	3,*N*4-ethenoC (3,*N*4-etheno-2′-deoxycytidine-5′-monophosphate)	Vinyl chloride metabolites Chloroethylene oxide Chloroacetaldehyde
	1,*N*2-ethenoG (1,*N*2-etheno-2′-deoxyguanosine-5′-monophosphate)	Vinyl chloride metabolites Chloroethylene oxide Chloroacetaldehyde β-Carotene oxidation products
	1 mA (1-methyl-2′-deoxyadenosine-5′-monophosphate)	Alkylating agents
	1 mG (1-methyl-2′-deoxyguanosine-5′-monophosphate)	Alkylating agents
	3 mC (3-methyl-2′-deoxycytidine-5′-monophosphate)	Alkylating agents
	3 mT (3-methyl-2′-deoxythymidine-5′-monophosphate)	Alkylating agents
Mismatch repair	Base mismatch	Polymerase mistake Spontaneous deamination Homologous recombination
	Small deletion loop	Polymerase mistake
	Large deletion loop	Polymerase mistake
	Large insertion loop	Polymerase mistake
	Small insertion loop	Polymerase mistake
Base excision repair	Base mismatch (base mismatch)	Polymerase mistake Spontaneous deamination Homologous recombination
	Single-strand break (single-stranded DNA break)	UV radiation Enzymatic cleavage Ionizing radiation
	Nick (nick)	Enzymatic cleavage
	AP site (apurinic site)	Spontaneous Unstable adducts Base excision repair
	dU (2′-deoxyuridine-5′-monophosphate)	Base excision repair Spontaneous deamination
	Thymidine glycol (5,6-dihydroxy-5,6-dihydrothymidine-5′-monophosphate)	UV radiation Reactive oxygen species
	3 mA (3-methyl-2′-deoxyadenosine-5′-monophosphate)	Alkylating agents
	3 mG (3-methyl-2′-deoxyguanosine-5′-monophosphate)	Alkylating agents
	7 mA (7-methyl-2′-deoxyadenosine-5′-monophosphate)	Alkylating agents
	8-oxoG (8-oxo-2′-deoxyguanosine-5′-monophosphate)	Reactive oxygen species
	FapyA (4,6-diamino-5-formamidopyrimidine-2′-deoxynucleoside-5′-monophosphate)	Reactive oxygen species Ionizing radiation
	FapyG (2,6-diamino-4-hydroxy-5-formamidopyrimidine-2′-deoxynucleoside-5′-monophosphate)	Reactive oxygen species Ionizing radiation
	7 mG (7-methyl-2′-deoxyguanosine-5′-monophosphate)	Alkylating agents
Nucleotide excision repair	6-4PP (dinucleoside monophosphate 6-4 photoproduct)	UV radiation
	CPD (cyclobutane pyrimidine nucleoside phosphate dimer)	UV radiation
	Bulky adduct	Large polycyclic hydrocarbon
Homologous recombination repair	DNA gaps; DNA double-stranded breaks (Dsbs); DNA interstrand crosslinks	Ionizing radiation, chemical agents, ultraviolet light
Non-homolog end-joining	Partially single-stranded DNA; double-stranded breaks	Enzymatic digestion

In mammals, the mechanism of DNA damage response and repair has been well studied because of its role in the initiation of cancers and its applications in cancer therapeutics (Tian et al., 2015). In plants, the DNA damage response is understudied but over the last decade has attracted enormous attention largely because of its consequences on the growth and development of plants (Manova and Gruszka, 2015). Plants exposed to excess DNA damage displayed a significant reduction in productivity and crop yield. It appears that the core components of the DNA damage response pathway are similarly organized in plants. Orthologous genes exist for master DNA damage response genes such as ataxia telangiectasia mutated (ATM) (Kurzbauer et al., 2021), ATM and Rad3 related (ATR), and meiotic recombination 11 (MRE11)–radiation-sensitive 50 (RAD50)–Nijmegen breakage syndrome 1 (MRE11-RAD50-NBS1) (MRN) complex (Cools and De-Veylder, 2009). The deletion of ATM and ATR in *Arabidopsis thaliana* presented no discernible phenotype *per se*. However, these plants are sensitive to DNA damaging agents such as aphidicolin, radiations, and alkylating agents. Furthermore, similar to mammals the activation of ATR and ATM is dependent on the MRN complex because the mutants of *rad50* and *mre11* are unable to activate ATR and ATM. Moreover, *rad50* and *mre11* mutants are sterile, indicating the inability of these plants to repair DNA damages affecting their ability to reproduce by either accumulation of mutations in meristem or by an unknown essential function in meiosis during gamete formation (Amiard et al., 2010). Furthermore, *ku80* mutants exhibited increased homologous recombination when exposed to increased stress conditions (Yao et al., 2013). Likewise, increased expression of DNA Pol lambda was observed in plants treated with excess hydrogen peroxide and sodium chloride (Roy et al., 2013). Taken together, these observations indicate that DNA damage response pathways are critical for the growth and development of plants by preventing the accumulation of mutations.

Plants are constantly exposed to adverse environmental settings such as heavy metals, drought, ultraviolet (UV) light, heat, lack of nutrients, and changing temperatures. Because of the sessile and autotrophic nature of the plant life cycle, they are unable to evade and escape these stressful conditions. For instance, the autotropic trait necessitates them to harness the sunlight for the production of food at the expense of exposure to UV light, resulting in the formation of toxic cyclobutane dimers in DNA (Dany et al., 2001). The photosynthetic and metabolic processes result in significant production of metabolic byproducts including reactive oxygen species (ROS) (Tuteja et al., 2009; Li et al., 2019). Production of ROS triggers single- and double-stranded breaks (SSBs and DSBs) in the DNA either directly through destruction of bases or modifications of bases. In some crop plants, oxidative stress imbalances ROS production and consequently promotes developmental defects and growth reduction (Rybaczek et al., 2021). This results in a significant decrease in plant productivity and crop quality. However, to prevent the toxic effects of ROS, plants normally keep a balance between the generation of free radicals and their eradication through the antioxidant system formed by superoxide dismutase, catalase, and ascorbate peroxidase (Li et al., 2019; Wang et al., 2019). These enzymes are vital for limiting the cellular accumulation of ROS. For instance, the mutants of *apx1* and *cat1* exhibit increased DNA damage demonstrating that ROS production has direct effects on the stability of plant DNA (Vanderauwera et al., 2011; Hu et al., 2016). Taken together, these observations underline the importance of DNA repair pathways for the prevention and accumulation of mutations on exposure to adverse environmental conditions. In exceptional cases, the mutations accumulate at an enormous rate upon many cell divisions and generations, separating one generation from the next affecting the plant viability. For instance, 6-year-old *Crepis tectorum* seeds showed reduced germination and a wide range of developmental abnormalities in the seedlings and mature plants (Navashin and Shkvarnikov, 1933). The phenotypic effects were exacerbated when seeds were stored at elevated temperatures. The mutant phenotypes from the plant phenocopies X-ray treated cells indicating accumulation of DNA damages in these seeds (Navashin and Shkvarnikov, 1933; Bray and West, 2005). Besides, the exposure of cereals and *Arabidopsis* to severe DNA damage results in DNA duplication without the ensuing cell division producing polyploid cells. The production of polyploid cells signifies permanent differentiation of cells (Galbraith et al., 1991). However, the same phenomenon of re-replication and severe DNA damage in meristems promotes cell death to avoid the transfer of these DNA damages to the next generation. Therefore, it appears that maintenance of genetic integrity is key to the survival of plants and for the transfer of accurate genetic information to subsequent generations. Surprisingly, despite the elevated exposure to DNA-damaging agents, it appears that the frequency of the mutation rate in plants is very low. Thus, plants must actively engage numerous genes in different DNA repair pathways to protect DNA from endogenous and exogenous stress (Table 2). In this review, we will summarize these complex mechanisms by which plants repair their DNA from severe exposure to biotic and abiotic stress.

**TABLE 2:** List of key genes that play vital roles in different DNA repair pathways.

Repair pathway	Genes	References
Direct reversal repair	ALKBH2 alkB, alkylation repair homolog 2 (*Escherichia coli*)	Duncan et al., 2002; Yang et al., 2008; Lenz et al., 2020; Toh et al., 2020
	ALKBH3 alkB, alkylation repair homolog 3 (*E. coli*)	Duncan et al., 2002; Yang et al., 2008; Fedeles et al., 2015; Lenz et al., 2020
	MGMT *O*-6-methylguanine-DNA methyltransferase	Tano et al., 1990; Mitra and Kaina, 1993; Ibrahim Al-Obaide et al., 2021
	PHR	Husain and Sancar, 1987; Li and Sancar, 1990; Sancar, 2016
	ADA	Jeggo, 1979; Shevell and Walker, 1991; Mielecki and Grzesiuk, 2014
Mismatch repair	EXO1 Exonuclease 1	Wilson et al., 1998; Lee et al., 2002; Sertic et al., 2020
	MLH3 mutL homolog 3 (*E. coli*)	Lipkin et al., 2000; Hawken et al., 2010; Hayward et al., 2020
	PMS1 PMS1 postmeiotic segregation increased 1	Hong et al., 2010; Li et al., 2020
	POLD1 Polymerase (DNA directed), delta 1, catalytic subunit 125 kDa	Dresler et al., 1988; Tsurimoto et al., 2005; Rytkonen et al., 2006; Nichols-Vinueza et al., 2021
	POLE Polymerase (DNA directed), epsilon	Rytkonen et al., 2006; Ewing et al., 2007; León-Castillo et al., 2020
Base excision repair	APEX1 APEX nuclease (multifunctional DNA repair enzyme) 1	Demple et al., 1991; Beernink et al., 2001; Coughlin, 2019;
	APEX2 APEX nuclease (apurinic/apyrimidinic endonuclease) 2	Rual et al., 2005; Burkovics et al., 2006; Briggs et al., 2010; Mengwasser et al., 2019
	FEN1 Flap structure-specific endonuclease 1	Murray et al., 1994; Zheng et al., 2008; Lu et al., 2020
	HUS1 HUS1 checkpoint homolog (*Schizosaccharomyces pombe*)	Volkmer and Karnitz, 1999; Liu C. Y. et al., 2010; Zhou et al., 2019
	MBD4 Methyl-CpG binding domain protein 4	Hendrich and Bird, 1998; Screaton et al., 2003; Sannai et al., 2019
	MPG *N*-methylpurine-DNA glycosylase	Miao et al., 2000; Ewing et al., 2007; Ryu et al., 2020
	NEIL1 Nei endonuclease VIII–like 1 (*E. coli*)	Das et al., 2007; Sengupta et al., 2018; Saini et al., 2020
	OGG1 8-Oxoguanine DNA glycosylase	Radicella et al., 1997; Lindahl and Wood, 1999; Ewing et al., 2007; Miglani et al., 2021
	PARP1 Poly(ADP-ribose) polymerase 1	Dantzer et al., 1998; Kanno et al., 2007; Wong et al., 2009; Lavrik, 2020
	PNKP Polynucleotide kinase 3 and phosphatase	Jilani et al., 1999; Karimi-Busheri et al., 1999; Kalasova et al., 2020
	RAD1 RAD1 homolog (*S. pombe*)	Parker et al., 1998; Zou and Elledge, 2003; Huangteerakul et al., 2021
Nucleotide excision repair	DDB1 Damage-specific DNA-binding protein 1	Keeney et al., 1993; Marini et al., 2006; Kim et al., 2016
	ERCC6 Excision repair cross-complementing rodent repair deficiency, complementation group 6	Selby and Sancar, 1997; Thorslund et al., 2005; Fousteri et al., 2006; Faridounnia et al., 2018; Apelt et al., 2020
	ERCC8 Excision repair cross-complementing rodent repair deficiency, complementation group 8	Henning et al., 1995; Selby and Sancar, 1997; Groisman et al., 2003; Fousteri et al., 2006; Lu et al., 2018; Moslehi et al., 2020
	MFD Mutation frequency decline	Selby et al., 1991; Oller et al., 1992; Martin et al., 2019; Leyva-Sánchez et al., 2020
Homologous recombination repair	EME1 Essential meiotic endonuclease 1 homolog 1 (*S. pombe*)	Briggs et al., 2010; Liu Y. et al., 2010; Wang et al., 2016
	FANCA Fanconi anemia, complementation group A	Kupfer et al., 1997; Bailey et al., 2010; Román-Rodríguez et al., 2019
	MRE11 Meiotic recombination 11 homolog A (*Saccharomyces cerevisiae*)	Paull and Gellert, 1998; Gatei et al., 2000; Lee and Paull, 2005; Chansel-Da Cruz et al., 2020
	RAD50	Bhaskara et al., 2007; Ghosal and Muniyappa, 2007; Chansel-Da Cruz et al., 2020; Völkening et al., 2020
Non-homologous end-joining	DCLRE1C DNA cross-link repair 1C (PSO2 homolog, *S. cerevisiae*)	Ma et al., 2002; Briggs et al., 2010; Liu Y. et al., 2010; Richter et al., 2019
	NHEJ1 Non-homologous end-joining factor 1	Ahnesorg et al., 2006; Buck et al., 2006; Esmaeilzadeh et al., 2019
	XRCC6 X-ray repair complementing defective repair in Chinese hamster cells 6	Cooper et al., 2000; Kim et al., 2008; Balinska et al., 2019
	YKU80	Ruan et al., 2005; Sabourin et al., 2007; Carballar et al., 2020
		

## DNA Repair Pathways

The integrity of DNA is under constant assault from endogenous and exogenous DNA-damaging factors including radiations, chemical mutagens, or spontaneously arising mutations. However, it appears that regardless of these assaults on DNA, the rate of mutation is exceptionally low because of the efficacy with which these alterations are fixed. To date, several pathways are known for repairing DNA damages; however, a few general assumptions can be made about these DNA repair mechanisms. First, most DNA repair pathways require a template strand for copying information into the damaged strand. The second general feature of DNA repair is the redundancy in repairing these damages, implying that a particular DNA error can be repaired by more than one repair pathway. The redundancy increases the likelihood of DNA repair and partly guaranteeing that practically almost all errors are corrected. At least five major DNA repair pathways viz. base excision repair (BER), nucleotide excision repair (NER), mismatch repair (MMR), homologous recombination repair (HRR), and non-homologous end-joining (NHEJ) repair are active throughout different stages of the cell cycle, allowing the cells to repair the DNA damage (Chatterjee and Walker, 2017). Direct chemical reversal and interstrand crosslink (ICL) repair pathways may also be exploited to clear unique lesions. These repair mechanisms are important for the genetic stability of cells. In this section, we will discuss general DNA repair mechanisms by which plants repair diverse kinds of DNA insults.

### Direct Reversal Repair

Direct reversal repair (DRR) removes certain DNA and RNA modifications, without excision, resynthesis, or ligation (Ahmad et al., 2015). It is an error-free repair pathway that retains the original genetic information because it does not involve the breaking of the phosphodiester backbone. To date, three major DRR mechanisms have been identified: (i) photoreactivation repair, (ii) direct DNA repair by alkyltransferase, and (iii) direct DNA repair by AlkB family dioxygenases (Yi and He, 2013).

#### Photoreactivation Repair

**FIGURE 1:**
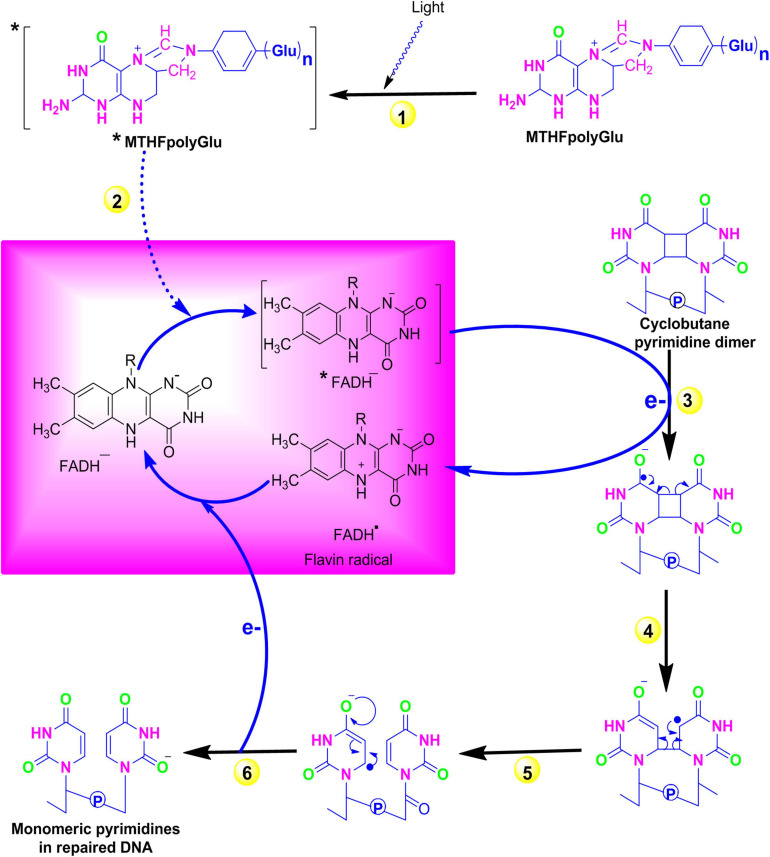
Repair of pyrimidine dimers with photolyase. (1) A blue-light photon is absorbed by the first chromophore MTHFpolyGlu, which functions as a photoantenna. (2) The electron from the excited MTFH* is then transferred to second chromophore FADH^–^. (3) The excited electron from *FADH^–^ is then transferred to pyrimidine dimer and converts it into pyrimidine monomers. (4–5) Electronic rearrangement restores the monomeric pyrimidines, and (6) the electron is transferred back to the flavin radical to regenerate FADH^–^. Source: Figure adapted and modified from Bray and West (2005). [MTFH (*N*5, *N*10 methenyl-tetrahydro folate); FADH^–^ (flavin adenine dinucleotide)].

The exposure of organisms to sunlight in the blue or UV-A spectrum results in the formation of cyclobutane pyrimidine dimers (CPD) such as thymidine–thymidine dimers. However, a process known as photoreactivation significantly decreases the biological consequences of these UV radiations by repairing these damages. A class of enzymes called photolyases specifically binds to these CPDs and directly reverses this damage in an error-free manner. Instead of removing the DNA-damaged region, photoreactivation reverses DNA damage to its original form in an error-free manner. In early life forms, it is believed to be the first evolved DNA repair mechanism and is still preserved in diverse species such as bacteria, yeast, plants, and animals (Lucas-Lledó and Lynch, 2009). In *Escherichia coli* energy derived from blue spectrum, light is absorbed by chromophores [*N*5, *N*10 methenyl-tetrahydro folylpolyglutamate and flavin adenine dinucleotide (MTHFpolyGlu and FADH^–^)] followed by sequential electron transfer from FADH to pyrimidine dimer. Finally, electronic rearrangement generates an unstable dimer radical that hydrolyses to yield the monomeric pyrimidines (Figure 1). Plants that are specialized in selectively reversing 6-4 photoproducts (6-4 PPs) or CPD, two distinct forms of photolyase enzymes such as 6-4 photolyase and class II photolyase, have been identified. These photolyases repair the lesions by binding at their respective DNA-damaged site in a light-independent manner and obtaining energy from the blue or near UV-A spectrum (Brettel and Byrdin, 2010). The photolyase genes are considered to be useful in modern agriculture to enhance the UV resistance and production of improved cultivars.

#### Direct DNA Repair by Alkyltransferases

**FIGURE 2:**
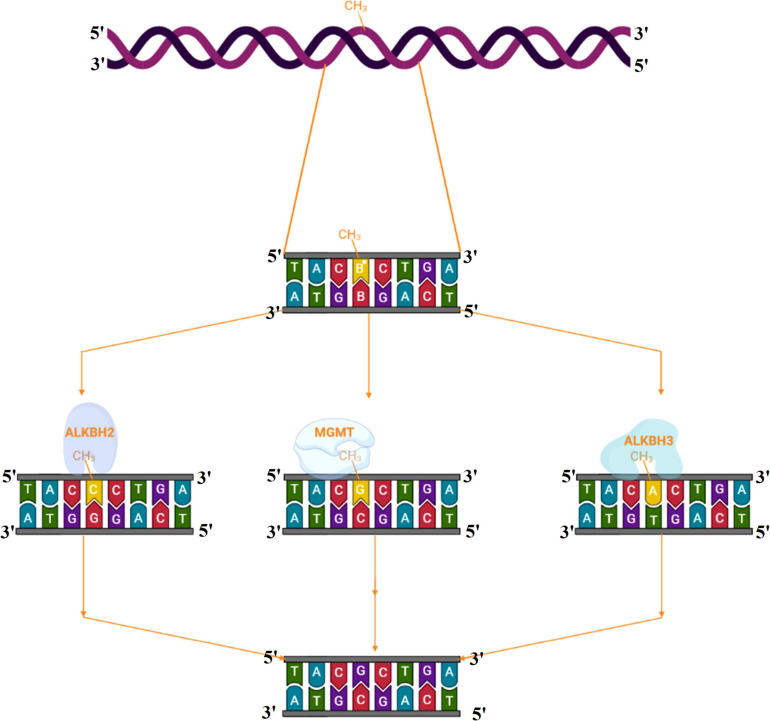
Direct reversal of N alkylated DNA bases by alkyltransferase and dioxygenase.

**FIGURE 3:**
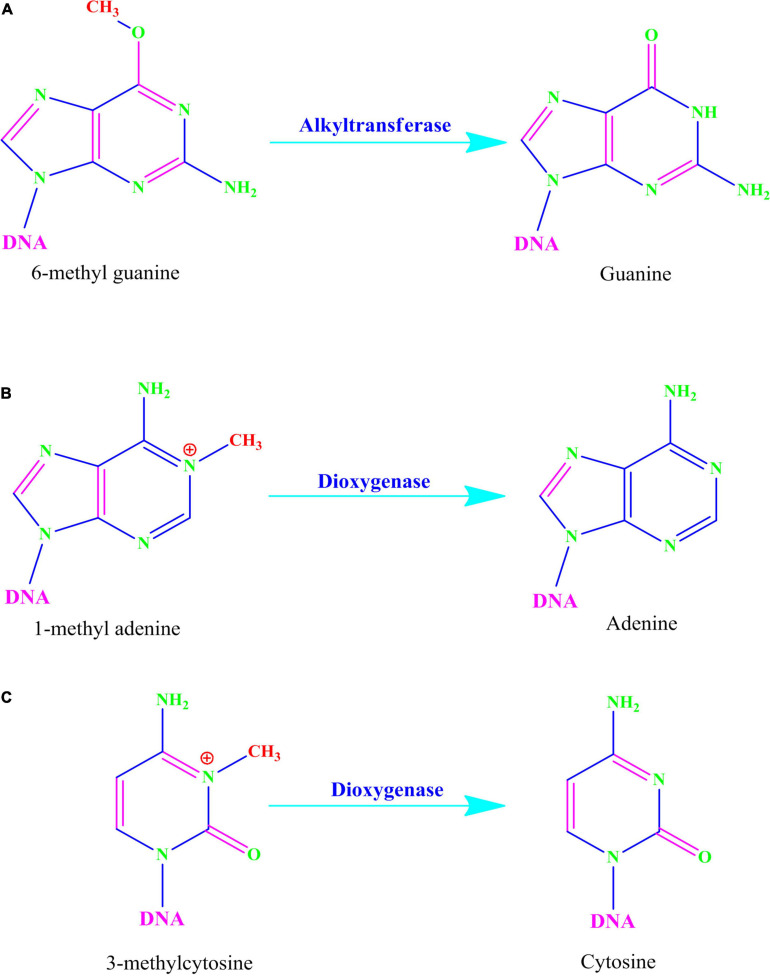
**(A)** Alkyltransferase mediated direct reversal of 6 methyl guanine to guanine. **(B)** Dioxygenase-mediated direct reversal of 1 methyl adenine to adenine. **(C)** Dioxygenase-mediated direct reversal of 3 methylcytosine to cytosine. Source: Figure adapted and modified from Yi and He (2013).

Alkylating agents react with the DNA and add alkyl groups preferably at *O*- and *N*- positions of nitrogenous bases. To combat the mutagenic effects of alkylating agents, organisms employ direct repair in which alkylated bases are screened followed by direct transfer of alkyl group from the nitrogenous base to the cysteine of an enzyme called O^6^-methylguanine-DNA methyltransferase (MGMT or AGT). MGMT binds in the minor groove of DNA, scans the DNA, repairs the alkylated bases, and therefore provides a quick repair for such DNA lesion. The MGMT protein, whose bacterial analog is called Ogt, specifically reverses guanine base methylation by removing methyl groups from the guanine (Pegg, 1990; Esteller et al., 2000; Ahmad et al., 2015). As each MGMT molecule can be used only once, the procedure is costly; the reaction is stoichiometric rather than catalytic (Ibrahim Al-Obaide et al., 2021). MGMTs are ubiquitous in both bacteria and higher organisms except fission yeast and plants (Pegg, 2011). The adaptive response in bacteria is a generic response to methylating agents that confers a degree of tolerance to alkylating agents by upregulating alkylation repair enzymes after prolonged exposure. The methylation of the bases cytosine and adenine by ALKBH2 and ALKBH3 is the DNA damage that cells can repair (Yang et al., 2008; Fedeles et al., 2015; Lenz et al., 2020; Figures 2, 3A). To date, no homologs for MGMT have been reported in plants; however, plants have evolved a mechanism for the removal of alkylated bases, and recent research implicates BER as a substitute for MGMT activity (Manova and Gruszka, 2015).

#### Direct DNA Repair by the AlkB Family Dioxygenases

AlkB family dioxygenases scan the genome and have the ability to alkylation lesions by flipping the alkylated or damaged base in both single-stranded DNA (ssDNA) and double-stranded DNA (dsDNA). In the event of oxidative dealkylation, the AlkB family dioxygenases require iron as cofactor and 2-oxoglutarate as cosubstrate for activation of dioxygen molecule for various oxidative reactions. The activated dioxygen molecule then oxidizes and removes the alkyl group from N1 adenine (1-methyladenine) or N3 cytosine (3-methylcytosine), to yield an unmodified base (Figures 2, 3B,C). The *E. coli* AlkB protein (EcAlkB) repairs the 1-methyladenine (1-meA) and 3-methylcytosine. ALKBH2 and ALKBH3 are the mammalian homologs of *E. coli* AlkB with ALKBH2 as the main repair enzyme for 1-meA (Yi and He, 2013). Plants have also evolved an adaptive mechanism that is similar to other eukaryotes to repair alkylated nitrogenous bases. Meza et al. (2012) have reported several AlkB homologs such as AT2G22260, which revealed sequence similarity to both ALKBH2 and ALKBH3 in *A. thaliana*. The *Arabidopsis* ALKBH2 protein also displayed *in vitro* repair activities on hydroxylated methyl and ethyl groups covalently linked to DNA. Furthermore, seedlings raised from alkbh2 knockout plants developed abnormally when grown in the presence of methyl methanesulfonate (MMS).

### Mismatch Repair

**FIGURE 4:**
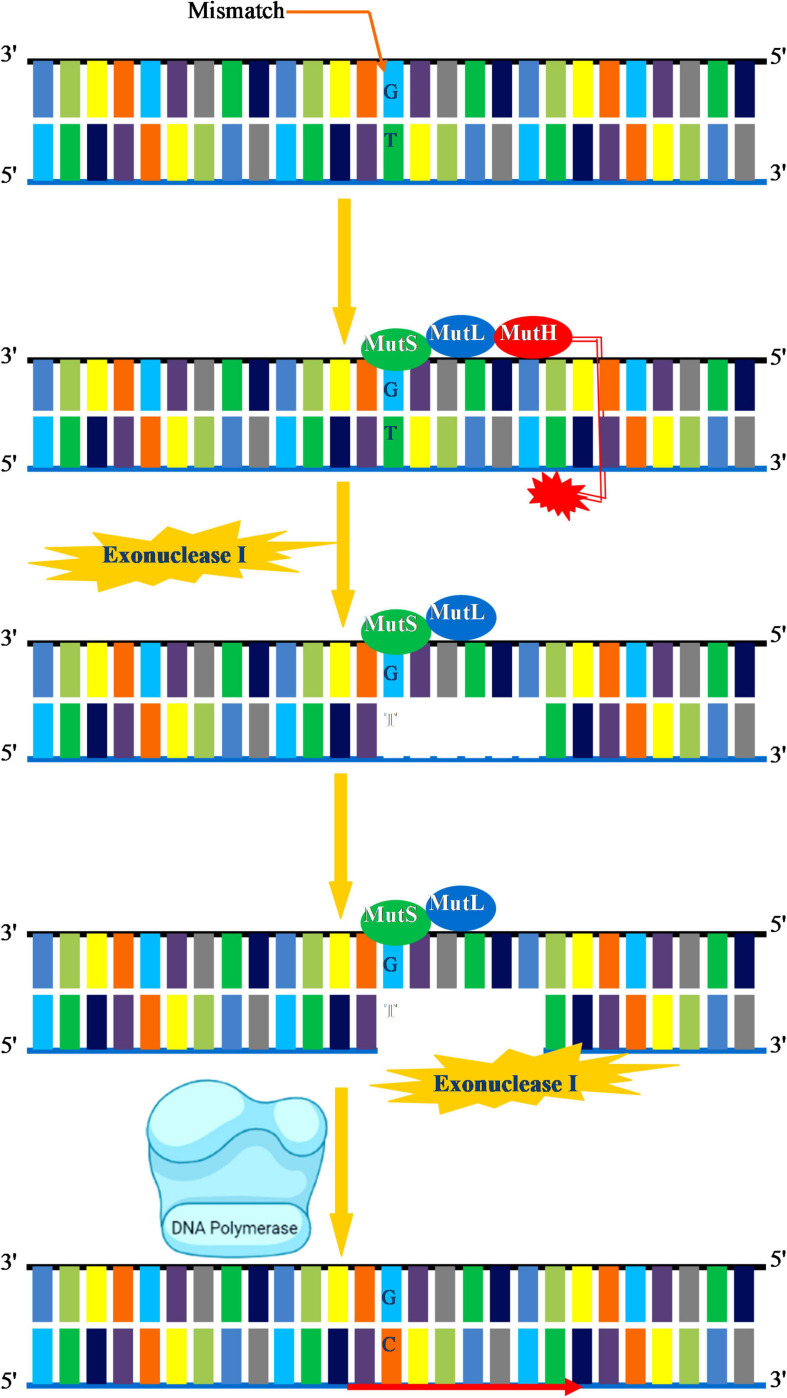
Mismatch repair. A G-T mismatch is recognized by MutS in association with MutL. MutH cleaves in the vicinity of mismatch. Exonuclease I initiates removal of DNA segment containing the incorrect base DNA. Exonuclease I completes the removal of damaged DNA. DNA polymerase III then synthesizes the new DNA and fills the gap.

DNA replication–mediated errors that escape fraying by the exonuclease activity of the DNA polymerase are corrected via an MMR system. In the MMR system, specific enzymes excise the newly incorporated incorrect nucleotide and replace it with the correct nucleotide. The key biological function of MMR system is to correct errors introduced during DNA replication. Besides, MMR is actively involved in the repair of mispaired intermediate bases, insertion–deletion, loops, elimination of unnecessary heteroduplexes, psoralen-induced ICLs, and oxidative DNA damage (Manova and Gruszka, 2015). Overall, MMR enables the cell to preserve genome integrity by increasing the DNA replication fidelity, decreases the frequency of mutations, and regulates the dynamics of short repetitive sequences, homologous recombination, and normal meiosis (Spampinato et al., 2009). MMR is strongly conserved in all living species as an important protection mechanism for preserving genomic integrity, although certain differences within the kingdoms appear to exist. In prokaryotes, MMR is majorly carried about by the concerted action of three main enzymes mutator (Mut)S, MutL, and MutH that direct the recognition and removal of the mismatch. MutS recognizes a G-T mismatch followed by a cut near the mismatch by MutH. The region containing mismatch is removed by exonuclease I, and a new DNA segment is synthesized by DNA polymerase III to fill the gap (Figure 4). In eukaryotes, MMR machinery mainly consists of MutSα/β comprising of (MutS homologs) *MSH2*, *MSH3*, *MSH5*, and *MSH6*, and MutL homolog comprising of *MLH1*, *PMS1 (MLH2)*, *MLH3*, and *PMS2 (MLH4).* Plants have an additional MSH gene called *MSH7* (Culligan and Hays, 2000). The general mechanism by which MMR functions in eukaryotes begins by the recognition of the mismatch by MutSα/β followed by the incision of the nick by MutLα. This allows for the recruitment of exonuclease 1 (EXO1), replication protein A (RPA), and Pol δ for the replacement of specific DNA segments through strand displacement synthesis. The role of MMR factors during postreplicative and recombination MMR is well known in plants. MSH2 deficiency in *Arabidopsis* prevents homologous but enhances homologous recombination and microsatellite instability in germline cells (Leonard et al., 2003; Li et al., 2006), whereas MSH7 regulates meiotic recombination, and its downregulation impairs meiotic recombination and fertility in cereals (Lloyd et al., 2006; Lario et al., 2015).

### Excision Repair

Unlike photoreactivation, other DNA repair pathways do not undo the DNA damage directly but instead substitute the damaged DNA with an appropriate nucleotide. Excision repair involves the removal of the damaged nucleotide by dual incision of the DNA strand containing the lesion (Waterworth et al., 2019). The incision is made on both sides of the lesion, followed by repair using the intact strand as a template. A common four-step pathway is used by these repair mechanisms that include (1) the initial detection of the DNA damage, (2) excision of the damaged nucleotide by the incision of a nick and subsequent removal of the damaged nucleotide(s), (3) filling of the gap by DNA polymerase using the exposed 3-OH as primer, and (4) finally sealing of the nick by DNA ligase. The mechanisms that determine the distance of the nick from the damage and the subsequent removal of the incorrect nucleotide permit the classification of this type of repair into two types, BER and NER.

#### Base Excision Repair

**FIGURE 5:**
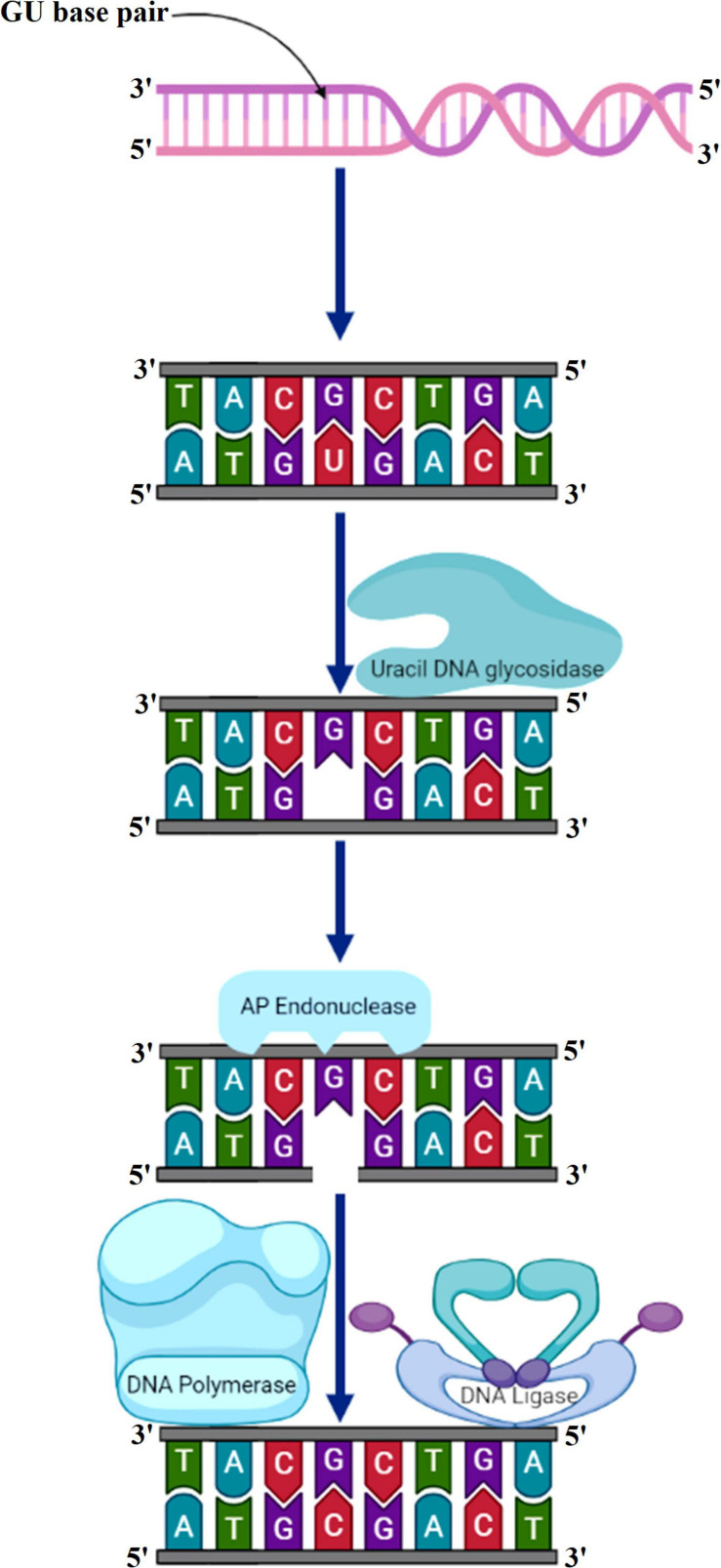
Uracil bases in DNA, formed by the deamination of cytosine, are excised and replaced by cytosine by the combined action of uracil DNA glycosylase, AP endonuclease, DNA polymerase, and DNA ligase. AP, apurinic/apyrimidinic.

**FIGURE 6:**
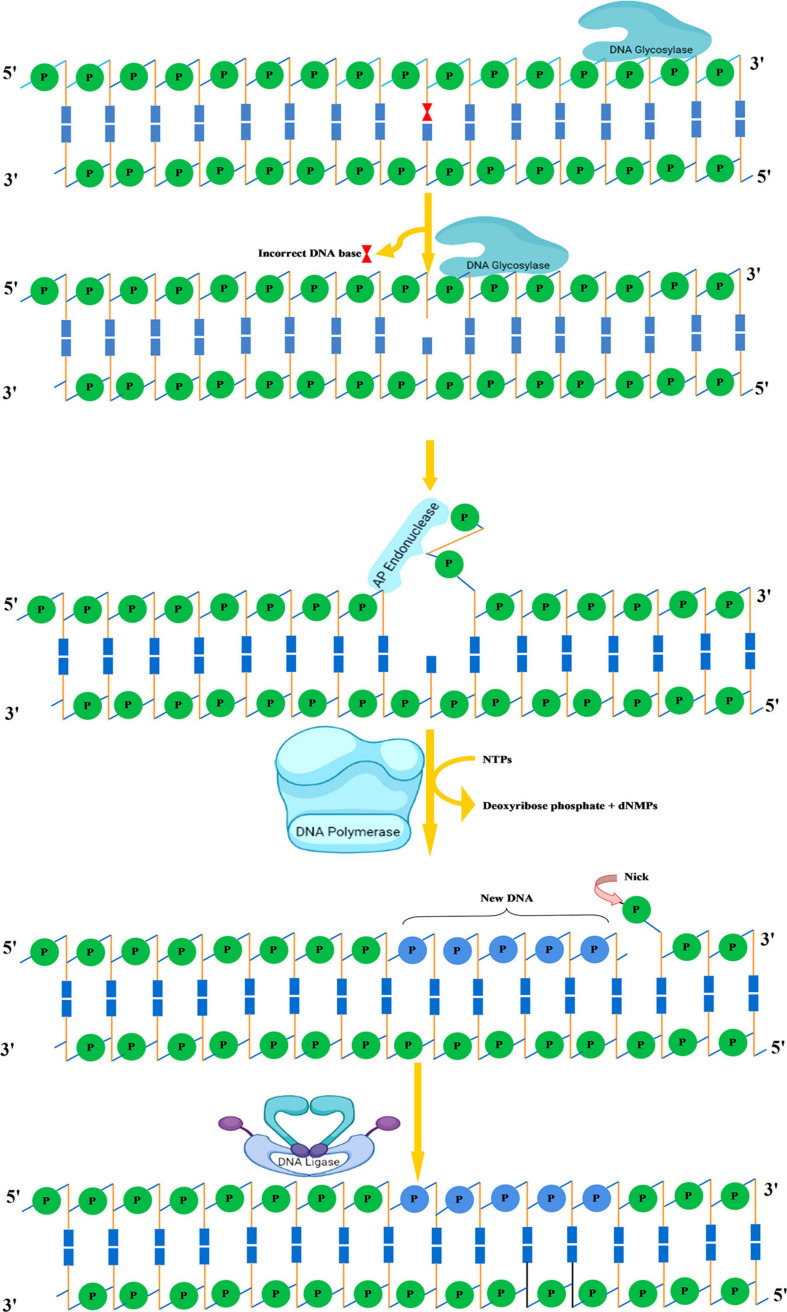
Base excision repair. Recognition followed by removal of damaged DNA base by DNA glycosylase resulting in the formation of AP site. An AP endonuclease nicks the phosphodiester backbone near the AP site. DNA polymerase I replaces the damaged portion with a new DNA. Finally, DNA ligase seals the nick. AP, apurinic/apyrimidinic.

The primary function of BER is to clear the genome of minute non-helix-distorting base lesions (Wallace, 2014). Bulky helix-distorting lesions are repaired by the associated NER pathway. BER acts on a variety of lesions including apurinic sites [apurinic/apyrimidinic (AP sites)], damaged and modified bases (Manova and Gruszka, 2015; Waterworth et al., 2019). Mechanistically in base-excision repair, the DNA glycosylase enzymes recognize and remove the modified/damaged bases from the DNA (Sakumi and Sekiguchi, 1990). This is followed by the removal of the nucleotide and a replacement of the polynucleotide strand. So far, in plants, several lesion-specific DNA glycosylases have been described; for instance, uracil glycosylase recognizes and removes uracil formed due to spontaneous deamination of cytosine (Figure 5). In *Arabidopsis*, whole-cell extract DNA containing uracil is repaired by the BER pathway in combination with uracil-DNA glycosylases. In particular, *in vitro* reconstitution of DNA repair reactions carried out with isolated cell extracts from *Arabidopsis* or other plants has been extremely helpful in identifying several structural and functional aspects of BER. Hypoxanthine, 3-methyladenine, 7-methylguanine, and other modified bases are recognized by other glycosylases. The first cloned plant DNA repair gene, *Arabidopsis* 3-methyladenine-DNA glycosylase, has been shown to eliminate MMS-induced DNA lesions (Santerre and Britt, 1994). Other bifunctional glycosylases, such as 8-oxoG DNA glycosylase/AP lyase, cut the DNA backbone on the 3′ side of the AP site followed by repair of 7,8-dihydro-8-oxoguanine (8-oxoG), a guanine oxidation product in *Arabidopsis* (Bray and West, 2005). More specifically, the lesion-specific DNA glycosylase hydrolyses the *N*-glycosidic bond linking the modified/damaged base to the 1′-carbon atom of deoxyribose sugar, without altering the DNA sugar-phosphate backbone. This results in the creation of an abasic site, which is then recognized by an AP endonuclease or AP lyase, which cuts the DNA backbone by cleaving the phosphodiester bond at the AP site (Figure 6). Subsequently, depending on the nature of the lesion and the enzyme involved, the repair response can either continue by “short” or “long” patch mechanisms. In mammalian cells, BER’s “short” mode exploits DNA polymerase β (Pol β), XRCC1 (X-ray repair cross-complementing protein 1), and LIG3α to repair a single-nucleotide gap The BER “long patch” removes 10 nucleotides surrounding the lesion and relies on the involvement of the DNA polymerase δ/ε-proliferating cell nuclear antigen-flap endonuclease 1 (δ/ε-PCNA-FEN1) complex. In plants, short-patch BER is an important DNA repair mechanism for uracil elimination in mitochondrial DNA (Boesch et al., 2009). The short-patch repair is less conserved because of the lack of plant homologs of DNA Pol β or DNA ligase III. Notably, considering the absence of Pol β and ligase III homologs in plants, all BER modes can occur after the initial incision stages, and the repair reactions are completed by the *Arabidopsis* DNA ligase 1 (AtLIG1) ligation (Cordoba-Cañero et al., 2009; Cordoba-Cañero et al., 2011). However, DNA polymerase λ in rice showed *in vitro* deoxyribose phosphate (dRP) lyase activity and sequence similarity with human Pol λ and therefore may be a substitute for Pol β (Uchiyama et al., 2004). Furthermore, XRCC1-like protein isolated from *Arabidopsis* is devoid of domains that mediate in the interaction of XRCC1 with Pol β, and LIG3α in mammals, however, possesses a conserved BRCT domain that mediates interaction with poly(ADP-ribose) polymerase (PARP) (Taylor et al., 1998; Uchiyama et al., 2004). There are at least two PARP activities in plants that may play role in BER and recombinational repair pathways (Amor et al., 1998; Babiychuk et al., 2001). It is pertinent to mention that SSBs in DNA during BER are inevitable intermediates and can act as substrates for nucleotide excision and recombination repair (Memisoglu and Samson, 2000). Several findings indicate that BER plays a critical role in repairing seed storage-induced oxidative DNA lesions in germinating embryos (Macovei et al., 2011; Cordoba-Cañero et al., 2014). Further understanding of these processes will help enhance the means of protecting seeds and discover new ways of preserving their capacity to germinate.

#### Nucleotide Excision Repair

**FIGURE 7:**
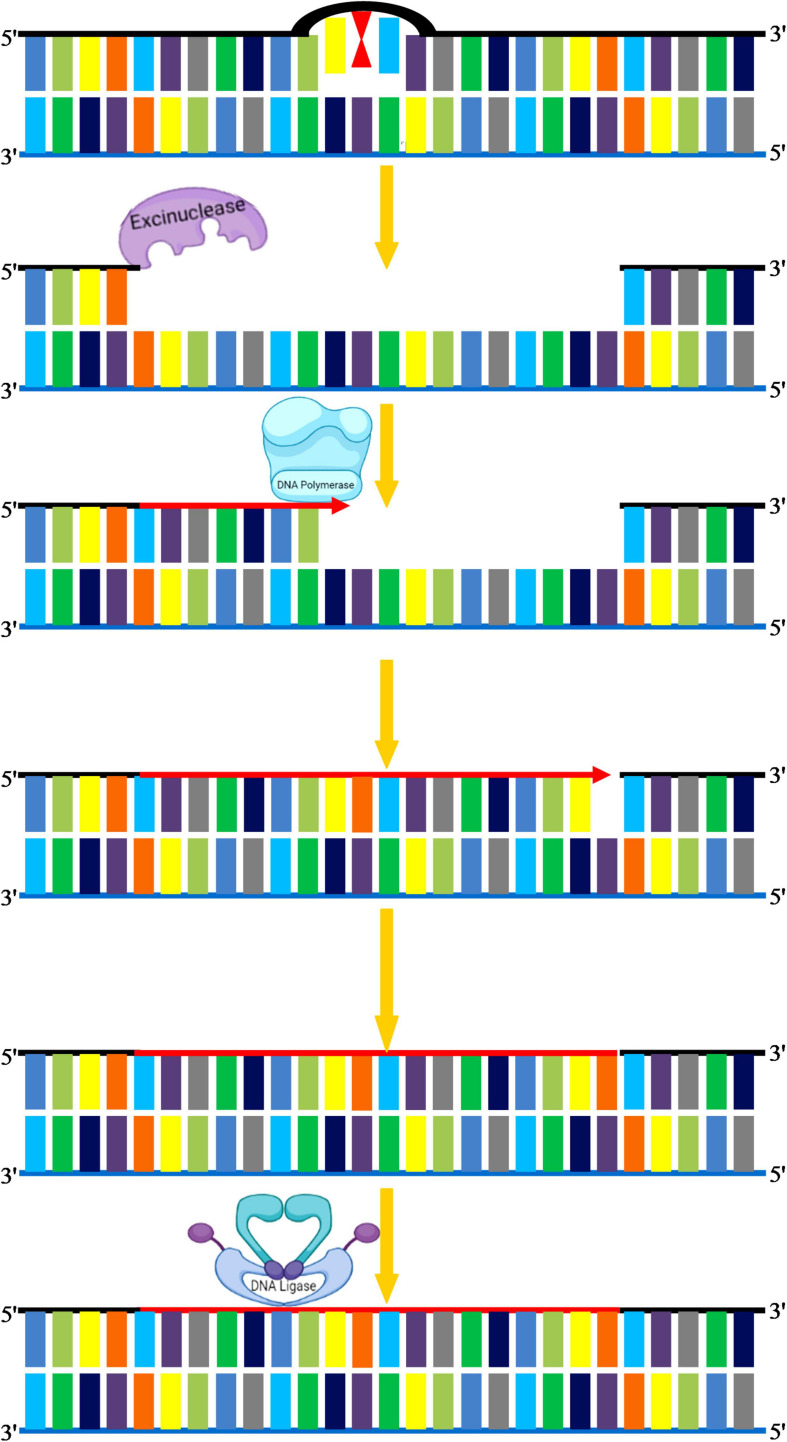
Nucleotide excision repair. NER eliminates these adducts by making an incision on both sides of adduct by excinucleases followed by the removal of this incised stretch of DNA. DNA polymerase I replaces the damaged portion with a new DNA. The gap is eventually filled by DNA ligase.

Nucleotide excision repair is used to repair bulky types of DNA damage, such as steric changes in DNA duplex structure or base dimers, in which an oligonucleotide of 30 bases is excised followed by DNA polymerase mediated resynthesis in the single-stranded region (Kusakabe et al., 2019; Ferri et al., 2020). This pathway can also recognize polymerase-blocking lesions using stalled RNA polymerase, which is then fed into the NER pathway (Waterworth et al., 2019). The minute details underlying mechanisms of NER have been explored by comprehensive studies in both simple and complex organisms. Mostly NER genes and associated repair proteins share a similar pattern of organization in both crop and model plants. In general, NER plays a critical role in corrections of structural alterations in regular DNA double-helix, and hence, it is conserved in both prokaryotic and eukaryotic organisms. For instance, UV-induced photo products such as pyrimidine dimers and 6-4 PPs that produce significant conformational changes in DNA are key substrates of NER. The serious human disorders caused by inborn genetic defects in NER proteins, such as xeroderma pigmentosum and Cockayne syndrome, demonstrate the significance of this repair process (Lehmann et al., 2018; Krokidis et al., 2020). NER eliminates these adducts by making an incision on both sides of the adduct followed by the removal of this incised stretch of DNA through a helicase (Marteijn et al., 2014). The gap is eventually filled by DNA Pol δ with the help of RPA, PCNA, and FEN1 (Figure 7). However, in plants, the homolog of human DNA Pol δ is not yet clear, and further research is required to demonstrate the enzyme that fills the gaps created by the removal of ssDNA on each side of the lesion. NER varies in two ways from BER: first, the diversity of DNA damage products recognized by the NER is strikingly large, and second, the repair complex initiates repair by creating nicks on the affected strand. These nicks occur at both 5′ and 3′ ends of the lesion at a particular distance, which is then excised as an oligonucleotide by the action of a helicase. Recent work suggests that DNA/RNA helicases can mitigate the negative effects of multiple abiotic stress factors (Gill et al., 2015). In eukaryotes, OsXPB2, a member of the strongly conserved helicase superfamily 2, is involved in DNA metabolism, such as transcription and repair (Umate et al., 2011). With differing efficiencies, the excision repair complex cleaves almost every DNA structure abnormally from very thin, non-distorting lesions (such as *O*^6^-methylguanine or abasic sites) to very bulky adducts (thymine-psoralen adducts or pyrimidine dimers). For every potential lesion, it is not feasible for a cell to create a particular repair enzyme; therefore, this pathway has evolved to deal with diverse kinds of damages. The efficacy of NER varies, depending on the nature of the DNA lesion and its genomic location. There are two separate NER subpathways: (a) global genomic repair (GGR) that repairs alterations in chromatin structure and DNA-associated proteins, (b) transcription-coupled repair (TCR) that eliminates transcription-locking lesions from the heavily expressed genes (Hanawalt, 2002). The two NER modes share the same repair proteins, however, differ primarily in sensing DNA damages. In higher eukaryotes, TCR recognizes stalled RNA Pol II complex on the transcribed strand after encountering DNA damage, and hence only this DNA strand is fixed quickly, whereas GGR recognizes damages on the coding strand that persist for longer durations (Tornaletti, 2005). GGR is dependent on xeroderma pigmentosum group C (XPC)/hHR23B complex stabilized by hCEN2 that mediates recognition of DNA damages (Thoma and Vasquez, 2003). Whereas TCR is independent of XPC is initiated on encountering stalled RNA polymerase II (Mu and Sancar, 1997). *Arabidopsis* deficient in AtCEN2 revealed reduced repair of UV-C–caused DNA damage *in vitro* (Molinier et al., 2004). As part of the *Arabidopsis* homolog of the human XPC protein (AtRAD4) recognition complex, the *A. thaliana* CENTRIN2 (AtCEN2) gene was implicated in the early stages of GGR, thereby modulating both NER and HRR. A relation between NER and HRR has also been shown to be an alternate mechanism for CPD repair in plants (Molinier et al., 2004; Liang et al., 2006). Hence, it can be concluded that several NER genes are related to factors involved in homologous recombination and photo repair in plants, and such a complex interplay of different DNA repair pathways could improve the plasticity and adaptability of the plant genome to a wide range of ecologies (Manova and Gruszka, 2015). For plants, the selective activity of excision repair mechanisms at the level of actively transcribed genes tends to be very important, and it may be useful to investigate the role of gene-specific repair in augmenting UV tolerance in crop species.

The key discrepancies in the mismatch, base excision, and nucleotide–excision repair mechanisms are in the identification and mode of excision of damaged nucleotide. In BER and MMR, a single nick is created in the sugar-phosphate backbone on one side of the damage, whereas in NER, nicks are made on both sides of the DNA damage. Furthermore, in BER, DNA polymerase displaces the old nucleotides when it extends the exposed 3’ end of the nick; in MMR, the old nucleotides are degraded, and in NER, nucleotides are displaced by helicase enzymes. DNA polymerase and ligase are used by all three pathways to fill in the gap created by the excision and for sealing the nick, respectively.

#### Homologous Recombination Repair and Non-Homologous End-Joining

The DNA repair mechanisms mentioned previously occasionally fail to completely repair the lesions, resulting in SSBs or DSBs. Additionally, these breaks can also be induced by the exposure of cells to exogenous agents such as ionizing radiation. DSBs are the most damaging of all the lesions, and a few unrepaired DSBs can lead to chromosomal fragmentation and even cell death (Dudáš and Chovanec, 2004; Sonoda and Hochegger, 2006). DSBs usually occur spontaneously within a cell, particularly during DNA replication and when the cell is under oxidative stress (Waterworth et al., 2019). These breaks in S-phase can obstruct the progression of the moving replication fork, resulting in a replication fork blockade (Hochegger et al., 2004). To circumvent the toxic effects of DSBs, organisms have evolved two pathways viz. homologous recombination and NHEJ for the repair of DNA breaks.

##### Homologous recombination repair

**FIGURE 8:**
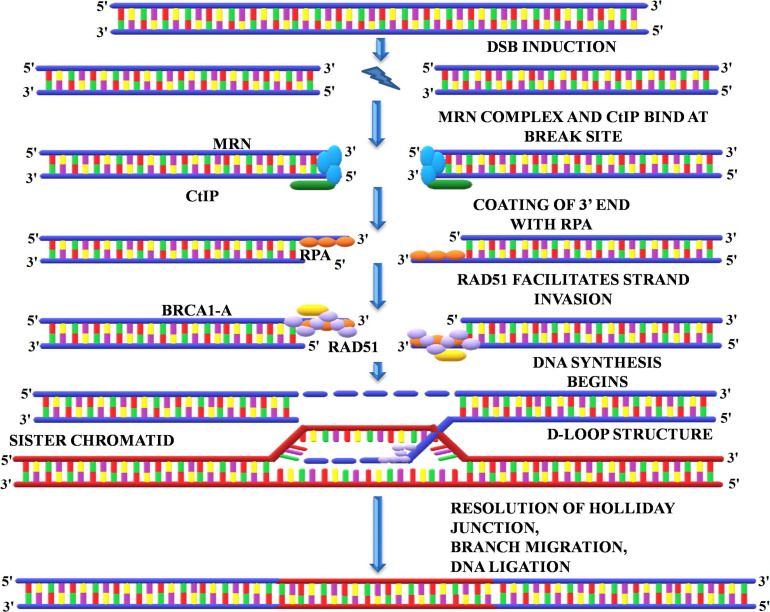
Homologous recombination repair. The HRR initiates with the recruitment of MRN, and CtIP complex at the repair site activates the kinases such as ATM and ATR. The MRN complex degrades the 3’ end followed by coating with replication protein A and binding of BRCA1/2, which subsequently recruits RAD51 and initiates DNA synthesis. RAD51 displaces the bound RPA and facilitates strand invasion into the homologous template that generates the D-Loop and Holliday junction, which are eventually resolved by resolvase. MRN, Mre11-Rad50-Nbs1; CtIP, carboxy-terminal interacting protein; ATM, ataxia telangiectasia mutated; ATR, ataxia telangiectasia mutated and Rad3 related; BRCA1/2, breast cancer1/2; RAD51, radiation sensitive 51.

A homologous recombination is a form of genetic recombination in which nucleotide sequences are swapped between two DNA molecules that are either related or identical. Cells normally use it to repair toxic double-strand breaks that occur on both strands of the DNA. During meiosis, the mechanism by which eukaryotes including animals and many plants make sperm and egg cells, homologous recombination creates new variations of DNA sequences. These new DNA combinations create genetic diversity in offspring, which allows populations to respond to changing conditions over time. The HRR pathway is a “flawless” DNA repair mechanism that repairs DSBs by using information encoded by homologous sequence. HRR is enabled by DSBs that occur inside replicated DNA (replication-independent DSBs) or at broken replication forks (replication-dependent DSBs). Production of the ends of the DNA double-strand break, homologous DNA pairing, and strand exchange, repair DNA synthesis, and resolution of the heteroduplex molecules are all part of HRR. To initiate the repair of the DSBs by homologous recombination, the DNA breaks must first be recognized, and an appropriate signal must be sent to the repair machinery for checkpoint activation. The repair initiates with the recruitment of the MRN complex at the site of DSBs (Charbonnel et al., 2010). MRN complex facilitates the recruitment of key regulators of DSB repair, protein kinases belonging to the phosphatidyl-inositol 3-kinase (PI3-kinase) family, ATM, and ATR (Figure 8). The MRN complex starts processing the DNA ends by the exonucleolytic degradation of the 3′ end followed by the activation of ATM/ATR that, in turn, phosphorylate the Sae2/CtIP and hundreds of other target protein involved in DSB repair and checkpoint activation (Charbonnel et al., 2010). The recruitment of these proteins is essentially required to generate the free 3′ ends and stabilization of DSBs. These 3′ overhangs produced by the excision of the 5′ end by MRN complex are coated with RPA to prevent its exonuclease-mediated degradation (Schmidt et al., 2019). This is followed by the binding o breast cancer 1/2 (BRCA1/2), which subsequently recruits RAD51 at the site of DSBs. RAD51 displaces the bound RPA and facilitates strand invasion into the homologous template (Mannuss et al., 2012). Next, the 3′ overhang coated with RAD51 locates the homologous sequence and invades the dsDNA by displacing the second strand of the template generating the “D-Loop” (displacement loop) (Dudáš and Chovanec, 2004; Mannuss et al., 2012; Ganai et al., 2016). After the formation of “D-Loop,” breaks can be either repaired by the synthesis-dependent strand-annealing (SDSA) model or double-strand break repair (DSBR) in which double Holliday junction (dHJ) intermediates are formed. The dHJ intermediates are resolved by resolvases that cut the crossed or non-crossed strands, resulting in the crossover or non-crossover products (Dudáš and Chovanec, 2004). The SDSA method uses the donor strand to fill the gap by using its sequence information, thus realigning the invasive strand to the original break site (Schmidt et al., 2019). In contrast to DSBR, the repaired end products always consist of non-crossovers. HRR uses the undamaged sister chromatid to restore the missing genetic information due to DSBs (Dudáš and Chovanec, 2004). As homologous sister chromatids are needed to repair the damage, it is therefore believed that HRR is only active during the S and G2 phases of the cell cycle (Davis and Chen, 2013). Interestingly, Gallego et al. (2005) identified six RAD51 paralogs in *Arabidopsis*, of which the expression of three is upregulated upon treatment with γ-irradiation. Thus, indicating that RAD51 paralogs play a central role in repairing γ-rays induced DSB through the HRR pathway.

##### Non-homologous end-joining

**FIGURE 9:**
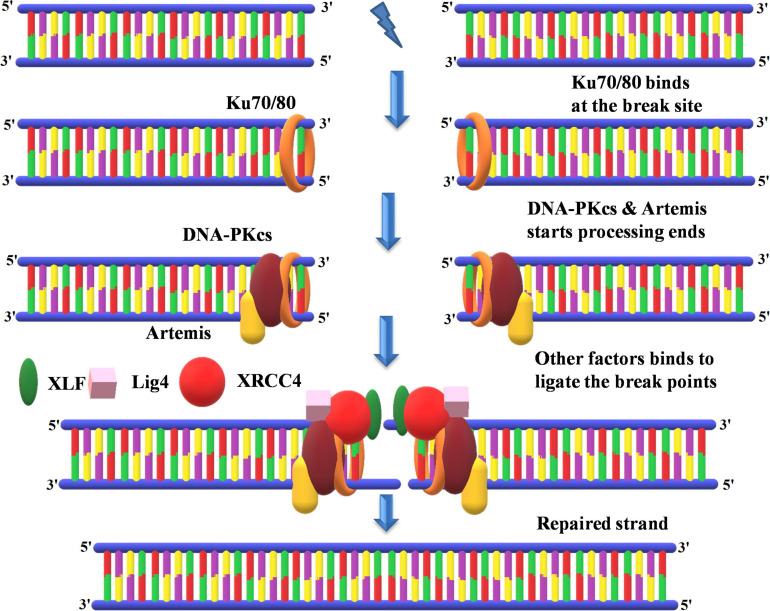
Non-homologous end-joining recombinational repair. KU70 and KU80 bind at the repair site followed by the processing of DNA ends by DNA-PKcs and Artemis. This is followed by the synthesis of new DNA in association with key proteins such as XLF, ligase 4, and XRCC4 at free DNA ends. KU70 and KU80, heterodimer protein with 70- and 80-kDa molecular weight; DNA-PKcs, DNA-dependent protein kinase, catalytic subunit; XRCC4, X-ray repair cross-complementing protein; XLF, XRCC4-like factor.

Non-homologous end-joining accounts for the most common form of DSB repair mechanism in plants (Puchta, 2005; Pannunzio et al., 2018). It involves the direct joining of two broken DNA ends. In comparison to homologous recombination, which involves a homologous sequence to guide repair, NHEJ directly ligates two ends without the need for a homologous sequence. NHEJ can be subdivided into two classes depending on the pathway used to repair the damage. The first one is KU-dependent classical/canonical NHEJ (c-NHEJ) repair, which encompasses direct ligation of the broken ends generally yielding error-free repair; however, occasionally small (usually less than a few nucleotides) insertions or deletions occur. In c-NHEJ, the KU heterodimer consisting of two subunits with 70- and 80-kDa molecular weight; i.e., KU70 (XRCC6) and KU80 (XRCC5) bind to the DSB to initiate the repair (Shen et al., 2017). As NHEJ involves rejoining the broken ends, the binding of KU not only prevents the damage of the free DNA ends but also assists in aligning the ends closer to each other (Mannuss et al., 2012). Subsequently, KU recruits other key proteins such as ligase IV, protein kinases C to repair the free DNA ends (Mannuss et al., 2012; Shen et al., 2017). In *Arabidopsis*, it was observed that AtKU70 and AtKU80 mRNAs increased threefold after induction of the DSBs (Mannuss et al., 2012). Thus, indicating that KU plays a crucial role in repairing DSB through the NHEJ pathway (Figure 9). Another NHEJ repair pathway works without the requirement of KU, and this pathway is referred to as backup-NHEJ pathway (b-NHEJ) or alternative NHEJ (Alt-NHEJ) or microhomology-mediated NHEJ because it acts in the absence of c-NHEJ. Very little is known about the mechanism of the b-NHEJ pathway, which involves multiple components such as polymerase (ADP-PARP1), but the function of PARP1 in c-NHEJ is not clear as it appears to be involved in a KU-dependent manner too (Shen et al., 2017). This pathway uses microhomologous sequences during the alignment of broken ends before ligating them together, thus resulting in deletions flanking either side of the original break. There are two conflicting reports regarding the repair of DSBs in plants. A study conducted in *A. thaliana* revealed that the predominant repair mechanism for DSBs is mediated by Alt-NHEJ exploiting DNA polymerase θ (PolQ) (Van Kregten et al., 2016). However, a second study reported that there are dissimilar mechanisms for the repair of DSBs in somatic and germ cells (Faure, 2021; Nishizawa-Yokoi et al., 2021). In the case of *A. thaliana* germ cells, the repair is completely dependent on Pol Q by Alt-NHEJ. However, the same authors in *A. thaliana* and rice somatic cells suggest the lack of an absolute requirement of Pol Q for the repair of DSBs revealing HRR is perhaps the predominant mechanism. Overall, these studies point toward the existence of a different mechanism for the repair of DSBs in plants. However, in mosses, the repair of DSBs predominantly occurs through HRR (Mara et al., 2019). *Pol q* deletion mutants do not show any developmental or genetic instability phenotype in mosses. Furthermore, these mutants showed the same sensitivity as wild type to DNA-damaging agents such as MMS, cisplatin UV rays except for bleomycin for which it was less sensitive than the wild type. These Pol Q mutants displayed enhanced HRR compared to wild type, indicating Pol Q acts as an inhibitor of the HR repair pathway. Taken together, these studies suggest that in mosses repair of DSBs predominantly occurs through HRR than Alt-NHEJ.

## Role of Small RNAs in DNA Damage Response

So far, we have explored the roles of various proteins in DNA damage response and maintenance of genome integrity. The vital roles of RNA in regulating DNA repair have started to emerge and reflect their importance in maintaining genome integrity by signaling DNA repair cascade via a mechanism not understood yet. However, recent evidence suggests a conserved and crucial role of RNA molecules, RNA processing enzymes, and other factors in DNA repair. It appears that most of the genome gets transcribed, but many of these transcripts do not code for proteins. These transcripts are called non-coding RNAs (ncRNAs), and some of these ncRNAs remain associated with chromatin in a sequence-specific manner to control many cellular pathways such as gene expression (di Fagagna, 2014). Recent studies about ncRNA reveal its additional role in refining the DDR. The structural integrity of DNA depends on small ncRNAs acting at the site of DNA damage. These small RNAs are recruited to the site of DNA damage and help transduce the signal for the recruitment of proteins at the site of DNA damage for accurate DNA repair.

The chemically induced replication stress led to an interaction between non-canonical small RNAs and DDR that led to subsequent production of small RNAs from actively transcribed ribosomal loci in *Neurospora crassa*, and this event was assisted by an ortholog of argonaute protein and RdRPs. These small RNAs were produced from the degradation of longer RNA species. The aberrant transcripts (“aRNA”) transcribed as a result of DNA damage are unresponsive to RNA polymerase inhibitors and are amplified by RdRPs and then processed into small RNA known as quelling-induced RNA (qiRNA). These qiRNAs then facilitate the degradation of aRNA, similar to the small interfering RNA (siRNA) amplification cycle (Schalk et al., 2017).

Wei et al. (2012) reported the production of diRNAs (DSB-induced small RNAs) in an *Arabidopsis* transgenic line. DSB repair through SSA (single-strand annealing) mechanism restores *b*-glucuronidase expression, which provides a visible and quantitative readout of DSB repair events (Wielgoss et al., 2013). The biogenesis of diRNAs requires the PI3-kinase ATR, RNA Pol IV, and Dicer-like proteins. Also, any kind of changes or directed mutagenesis in these proteins has resulted in a significant reduction in DSB repair efficiency, which confirmed the role of small RNA in DNA repair efficiency. As discussed in the above sections, UV radiations induce the formation of CPDs and 6-4 PP, which damage DNA structure and disturb cell/genome integrity by distorting regular DNA double-helical structure. However, plants have evolved a mechanism to escape and mitigate UV-induced irreversible DNA damage at their growing points. For instance, in UV-irradiated *A. thaliana*, the DNA damage-binding protein 2 (DDB2) and argonaute 1 (AGO1) form a chromatin-bound complex together with 21-nucleotide-long siRNAs, which perhaps assist in recognizing damage sites in an RNA/DNA complementary strand-specific manner. Synthesis of siRNA, which is associated with the PPs, involves the unusual concerted action of RNA polymerase IV, RNA-dependent RNA polymerase-2, and Dicer-like-4 (DCL4). Moreover, the association/dissociation of the DDB2-AGO1 complex with chromatin is under the control of siRNA abundance and DNA damage signaling, thus providing a view on the interplay between small RNAs and DNA repair recognition factors at damaged sites (Schalk et al., 2017).

## Scope of DNA Repair Mechanisms in Crop Improvement

Biotic and abiotic stresses frequently affect various developmental stages of crop plants and reduce their economical yield. Additionally, these stressful conditions also influence the efficiency of DNA repair pathways resulting in increased mutation frequency and genetic variability. Higher genetic variability in any species may evolve new phenotypes that can significantly enhance the adaptability to a range of ecologies (Wielgoss et al., 2013). DNA repair pathways have played an important role in induced mutagenesis as mutagens induce a wide range of DNA damages, which can have disastrous consequences on the integrity of the genome. However, some of these erroneous mutations can have beneficial consequences as well and are chosen by natural selection. These mutations have played an immense role in crop improvement programs by increasing genetic variability and developing new mutant varieties with improved traits within a short period, which can be further explored by the plant breeders (Oladosu et al., 2016). To date, it has made an immense contribution in the improvement of yield, maturity durations, and biotic and abiotic stress resistance and still utilized by plant breeders across the globe for crop improvement (Oladosu et al., 2016). Moreover, the improved mutant varieties play a vital role in crop biodiversity and offer useful breeding material for further crop improvement (Chaudhary et al., 2019; Raina et al., 2020).

Significant advancement in food production and quality has been recorded over the last six decades with the help of available genetic variation and diversity in crop plants. Although looking at the rising human population and reduced cultivable lands, further improvement in food production and nutritional quality is required in the near future. Expanding the knowledge of DNA repair processes in plants will possibly pave the way for interesting biotechnological applications aimed at improving stress tolerance in crops. Several researchers have reported the role of various enzymes and genes in DNA repair and subsequent productivity of plants.

Alterations in the expression pattern of genes have been reported to promote several beneficial activities in *Arabidopsis*. Kaiser et al. (2009) reported that the overexpression of photolyase enzyme may increase total biomass production under elevated UV-B radiation. Vanderauwera et al. (2007) demonstrated that reduced PARP levels in transgenic *Arabidopsis* led to enhanced tolerance to a wide range of abiotic stresses. Kimura and Sakaguchi (2006) reported the UV tolerance in *Arabidopsis* and rice by overexpression of the gene encoding the CPD photolyase enzyme. Similarly, the activity of helicases is usually up-regulated during stress conditions in plants. Vashisht and Tuteja (2006) demonstrated the overexpression of helicase enzyme in high salinity stress.

The disruption of MMR activities in plants through RNAi, CRISPR/Cas9, zinc-finger nucleases (ZFN), transcription activator–like effector nucleases (TALEN), or any other genetic engineering tools may perhaps create huge genetic variation and diversity as required for crop improvement. This phenomenon may generate novel plant types with desirable traits. The depletion of the nuclear-encoded DNA MMR protein MSH1 causes desirable and heritable changes in plant development. Several researchers reported that disruption of MSH1 genes in *Arabidopsis*, rice, potato, tomato, soybean, sorghum, and tobacco may drastically change their phenotypes and produce a wide range of novel plant types (Santamaria et al., 2014; Virdi et al., 2015; Rakosy et al., 2019; Jiang et al., 2020). A different spectrum of mutations gradually accumulates in MMR-deficient genotypes and increases generation after generation (Chao et al., 2008). However, stabilization of these mutations is quite complicated and still a big challenge to plant biologists. Stabilization can be achieved by bringing back active MMR proteins in the genetically reprogrammed plants or by crossing the mutant with their immediate parent. Moreover, the active MMR gene may stabilize the indels or mutations that occurred in the previous generation and produce genetically reorganized plants (Virdi et al., 2015; Yang et al., 2015).

## Site-Directed Mutagenesis

**FIGURE 10:**
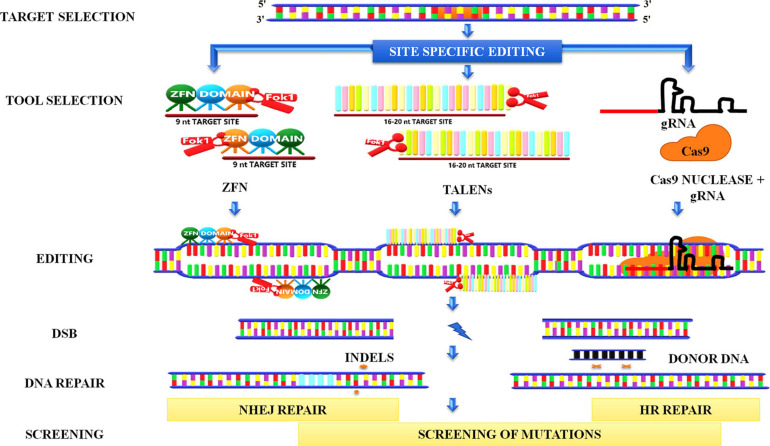
Genome editing. Site-directed genome editing involves the induction of site-specific double-stranded breaks in the genome followed by the recruitment of endonucleases such as ZFNs, TALENs, CRISPR-Cas9. ZFN recognizes nine nucleotide sites on binding and creates a break when two FokI monomers are in proximity to each other. TALENS also works in a similar manner. In the CRISPR system, a gRNA binds to the target site in the genomic region and forms a complex with Cas 9 nuclease to create a break. These breaks can further be repaired by NHEJ, which inserts indels in the sequence, or by HRR pathways in which homolog donor sequence could be used to modify the target sequence. ZFNs, Zinc-finger nucleases; TALENs, transcription activator–like effector nucleases CRISPR-Cas9, clustered regularly interspaced short palindromic repeats/CRISPR-associated protein 9; gRNA, guide RNA; FokI, a type IIS restriction endonuclease isolated from *Flavobacterium okeanokoites*; NHEJ, non-homologous end-joining.

Genome editing has emerged as one of the finest innovations in the field of plant biotechnology. The method encompasses the induction of site-specific DSBs by nucleases in the genome followed by exploitation of the repair of these breaks that lead to a generation of desired mutations. Smih et al. (1995) are the pioneers who induced DSBs in mammalian cells to study DNA repair by expressing I-SceI (intron-encoded endonuclease from *Saccharomyces cerevisiae*). Subsequently, various endonucleases such as meganucleases, ZFNs, transcription activator–like effector nucleases (TALENs), and clustered regularly interspaced short palindromic repeats/CRISPR-associated protein (CRISPR-Cas9) were used to induce site-specific DSBs in the genome (Figure 10).

Zinc-finger nucleases contain a DNA-binding domain through which they bind to DNA by recognizing three base pairs at the target site (Kim et al., 1996; Kumawat et al., 2019). To induce DSB, FokI, a type IIS restriction endonuclease isolated from *Flavobacterium okeanokoites*, must act as a dimer; therefore, each FokI monomer is attached to two DNA-binding ZFNs that recognize different DNA sequences (Townsend et al., 2005; Kumawat et al., 2019). When the monomers are closer to each other, FokI is activated and creates a DSB. Bibikova et al. (2001) used ZFNs to induce site-specific DSB in *Xenopus* oocytes that stimulate the HR repair pathway. Interestingly they also showed that targeted mutagenesis could be achieved by NHEJ as a result of ZFN-induced DSB in *Drosophila* (Bibikova et al., 2002). Later, Lloyd et al. (2005) utilized this technique to induce mutations at specific sites in *Arabidopsis*. Targeted mutations conferring herbicide resistance were achieved by altering the sequences of the endogenous acetohydroxyacid synthase (*SuRA* and *SuRB*) genes in the tobacco plant (Wright et al., 2009).

Transcription activator–like effector nucleases is another class of nuclease used for site-directed mutagenesis that focuses on a single nucleotide as opposed to three for ZFNs (Kim et al., 1996; Boch et al., 2009). The structural feature of the TALEN protein is unique in many ways, making it compatible with the design editing tool because it includes the nuclear location signal, N-terminal translocation signal, the acid activation domain, and the central repeat domain that binds DNA (Li et al., 2011a). Li et al. (2011b) designed hybrid TALEN to induce DSB in tobacco leaves.

CRISPR/Cas is the most promising and efficient genome-editing technique than the nucleases discussed above. CRISPR/Cas was discovered in bacteria or archaea as a type II prokaryotic adaptive immune system, which provides bacteria immunity against invading phages (Jinek et al., 2012; Kumawat et al., 2019). The mechanism of immunizing bacteria against viral attack starts with the incorporation of protospacer, which are small fragments of a foreign sequence in the host chromosome at the proximal end of the CRISPR array (Jinek et al., 2012). The protospacer consists of identical repeats, the transcription product of these repeats results in the generation of precursor CRISPR RNA (pre-crRNA). Later, enzymatic cleavage leads to the formation of crRNA, which has the ability to complementarily base pair with the protospacer sequence of the invasive viral target (Jinek et al., 2012; Kumawat et al., 2019). After recognition of target and complementary base pairing, Cas9 nuclease digests the target sequence and directs the silencing of viral sequences. In bacteria, there are three types of CRISPR/Cas systems known to date, viz. types I, II, and III. Type II system is most commonly used in genome editing. In the type II system, transactivating crRNA (tracrRNA), which is complementary to the pre-crRNA, in the presence of Cas9 tracrRNA helps in the maturation by processing with the ds-RNA–specific ribonuclease RNase III (Jinek et al., 2012; Kumawat et al., 2019). For efficient genome editing, single-guide RNAs (sgRNAs) are synthesized by combining the tracrRNA and crRNAs in which 5′ sequence of sgRNA binds to the target sequence and 3′ sequence binds to the Cas9 nuclease (Kumawat et al., 2019). The targeted mutagenesis by CRISPR/Cas9 is achieved by generating the sgRNAs complementary to the desired site, which allows binding of Cas9 to the desired site. The Cas9 enzyme subsequently cleaves the DNA at the desired site, resulting in the DSB, which is repaired by the HRR or NHEJ pathway leading to small indels. To confirm the role of KU in the NHEJ pathway in plants, Shen et al. (2017) utilized the CRISPR/Cas9 system to induce DSB in two genes, i.e., *Arabidopsis cruciferin 3* (CRU3) and protoporphyrinogen oxidase and observed larger deletions in mutants lacking KU.

## Future Directions and Concluding Remarks

The plant DNA damage response is evolving as a key process influencing plant growth and development in response to adverse environmental cues. The DNA damage response directly influences genome stability by preventing the accumulation of mutations within the organism. The literature discussed in this review reflects the dearth of data regarding the process of genome stability in plants compared to bacteria, yeast, and human. Given the climate change and the stress it imposes on plant growth and productivity, future research in this area will provide important insights into how plants maintain genome stability under stressful conditions. Characterizing various novel interactions between DNA repair proteins in response to stress will open new avenues for crop improvement. Furthermore, with the advent of CRISPR-Cas9 screens, it will be exciting to identify novel genes involved in DNA repair in plants not otherwise possible by classical genetics. Another promising line of research is to understand the link between DNA repair and chromatin dynamics. DNA repair proteins and processes require access to the DNA damage, which requires extensive chromatin remodeling and epigenetic modifications at the site of the DNA damage. It will be fascinating to uncover such modifications and further determine if such chromatin states are stable and heritable during stressful conditions. These heritable states will allow plants to acclimatize to such adverse environmental conditions. Future work would thus require understanding the mechanism of the initiation of these epigenetic states and designing assay systems that will allow us to study the heritable nature of these epigenetic states.

## Author Contributions

All authors wrote the initial draft with the following contribution—RG: introduction, future directions, and concluding remarks; AR and RL: DNA repair pathways; NR: recombinational repair; RS: role of small RNAs in DNA damage response; and PS: site-directed mutagenesis and scope of DNA repair mechanisms in crop improvement. After the initial draft was framed, AR and RG rewrote the review. SK throughout the process of writing contributed to the overall assessment of the manuscript.

## Conflict of Interest

The authors declare that the research was conducted in the absence of any commercial or financial relationships that could be construed as a potential conflict of interest.
